# Network pharmacology analysis and experimental verification reveal the mechanism of the traditional Chinese medicine YU-Pingfeng San alleviating allergic rhinitis inflammatory responses

**DOI:** 10.3389/fpls.2022.934130

**Published:** 2022-08-09

**Authors:** Zhen Liu, Qi Sun, Xinyue Liu, Zheying Song, Fei Song, Congxian Lu, Yu Zhang, Xicheng Song, Yujuan Yang, Yumei Li

**Affiliations:** ^1^Department of Otorhinolaryngology, Head and Neck Surgery, Yantai Yuhuangding Hospital, Qingdao University, Yantai, China; ^2^Shandong Provincial Clinical Research Center for Otorhinolaryngologic Diseases, Yantai, China; ^3^Clinical Medicine College, Weifang Medical University, Weifang, China; ^4^Second Clinical Medicine College, Binzhou Medical University, Yantai, China

**Keywords:** YPFS, hub genes, decursin, network pharmacology analysis, allergic rhinitis

## Abstract

YU-Pingfeng San (YPFS) can regulate inflammatory response to alleviate the symptoms of nasal congestion and runny rose in allergic rhinitis (AR). However, the mechanism of action remains unclear. In this study, 30 active ingredients of three effective herbs included in YPFS and 140 AR/YPFS-related genes were identified by database analysis. Gene ontology (GO) and Kyoto Encyclopedia of Genes and Genomes (KEGG) enrichment analysis showed that the targets were mainly enriched in immune inflammatory-related biological processes and pathways. Finally, three hub gene targeting epidermal growth factor receptor (EGFR), mitogen-activated protein kinase 1 (MAPK1), and protein kinase B1 (AKT1) related to YPFS and AR were identified by network pharmacology analysis. YPFS treatment decreased the expression of EGFR, MAPK1, and AKT1 in ovalbumin (OVA)-induced AR mice and impaired the production of inflammatory factors interleukin (IL)-4, IL-5, and IL-13, thus alleviating immunoglobulin E (IgE) production and the symptoms of scratching nose in AR. Through molecular docking analysis, we found that the active ingredients decursin, anomalin, and wogonin of YPFS could bind to EGFR, MAPK1, and AKT1 proteins. Moreover, decursin treatment impaired the expression of IL-4 and IL-5 in human PBMCs. These results suggested that YPFS could alleviate the AR inflammatory responses by targeting EGFR, MAPK1, and AKT1, showing the mechanism of action of YPFS in AR treatment.

## Introduction

Allergic rhinitis (AR) is an allergic disease of the nasal mucosa caused by a variety of factors. Clinical manifestations are characterized by nasal itching, sneezing, hypersecretion, and nasal mucosal swelling (Bousquet et al., 2020). AR is one of the most prevalent allergic diseases, with an estimated prevalence of 10–40% (Yan et al., 2021). AR affects the quality of life of patients and leads to increased personal and social medical costs. Currently, the treatment of AR mainly includes avoidance of exposure to allergens, drugs, surgery, and immunotherapy (Santos et al., 2015). Helper T cells (Th) 2 inflammation in the airway is characterized by induction of Th2 cytokines including interleukin (IL)-4, IL-5, and IL-13, leading to the production of immunoglobulin E (IgE) and inflammatory cell infiltration, which plays an important role in AR (Karta et al., 2016). In the pathological state of AR, the proportion of Th2 cells increases, causing an abnormal immune response, and reducing the Th2 response can reduce the inflammatory response of AR (Kappen et al., 2017). Many traditional Chinese medicines (TCMs) are used to treat AR, such as Shenqi and artemisia annua, which have a good therapeutic effect (Shao et al., 2017). In addition, it has been reported that YU-Pingfeng San (YPFS) is used frequently to treat allergic diseases, such as AR, asthma, and urticaria in modern medicine (Shen et al., 2014). However, the mechanism of action of YPFS in the treatment of AR is unclear.

YPFS, a TCM prescription, originated from The Danxi Heart Method written by Zhu Zhenheng in the Yuan Dynasty. YPFS is composed of *Astragali Radix* (Huangqi, HQ), *Atractylodis Macrocephalae Rhizoma* (Baizhu, BZ), and *Saposhnikoviae Radix* (Fangfeng, FF), at a content ratio of 3:1:1 (Xue et al., 2021). YPFS is recommended for the management of AR by the Chinese medicine clinical practice guideline (Luo et al., 2017). Chan et al. have reported that YPFS can reduce AR symptoms and enhance the quality of life (Chan and Chien, 2014). And Luo et al. reviewed 22 randomized controlled trials which showed that YPFS had an obvious effect on AR, and YPFS’s combination treatment seemed more beneficial (Luo et al., 2017). In addition, these 22 Randomized controlled trials also indicated that YPFS was well-tolerated for treating adult AR (Luo et al., 2017). All these studies suggested that YPFS is effective and safe for AR treatment in clinical. Many anti-rhinitis substances and their potential targets have been found in YPFS (Nikles et al., 2017); however, which active ingredients in them mainly function for AR treatment are not clear. Network pharmacology analysis is a new strategy to analyze the molecular regulation mechanisms of TCM active ingredients (Zhang et al., 2021). We aimed to explore the active ingredients of YPFS and their targets for the treatment of AR through network pharmacology analysis and *in vitro* and *in vivo* experiments to reveal the mechanism of YPFS against AR and provide potential targets for AR treatment.

## Materials and methods

### Screening of active ingredients in YU-Pingfeng San

The active ingredients of YPFS were predicted in the TCM System Pharmacological Database and Analysis Platform (TCMSP^1^). Those chemical compounds of YPFS with identifiable properties of absorption, distribution, metabolism, and excretion (ADME) and druggable properties, were selected as active components for further target prediction, with a cutoff of oral bioavailability of 30% and drug-likeness (DL) of 0.18 (Zhou et al., 2021).

### Searching for the targets of the active ingredients of YU-Pingfeng San

The three-dimensional structures of the identified compounds were obtained from the PubChem Database^2^ (Bahadur Gurung et al., 2022), the largest chemical compound information database in the world (Islam et al., 2021). The database of Swiss Target Prediction^3^ was used to predict the potential protein targets of the bioactive ingredients of YPFS, according to the three-dimensional structure of the compounds (Yang et al., 2020; Fan et al., 2021). All targets were combined for further study.

### Identification and functional analysis of the intersecting allergic rhinitis/YU-Pingfeng San-related genes

AR-associated genes were selected and combined from the Drug Bank Database^4^ the Gene Card Database^5^ and the Human Mendelian Genetics Online Database (OMIM^6^; Tekin et al., 2016). AR/YPFS-related genes were obtained by selecting the overlapping genes between AR-associated genes and the targets of active ingredients of YPFS using a Venn diagram.^7^

### Gene ontology and Kyoto Encyclopedia of Genes and Genomes pathway analysis

Gene ontology (GO) and Kyoto Encyclopedia of Genes and Genomes (KEGG) pathway enrichment analysis of AR/YPFS-related genes was performed to identify genes in the biological processes (BP), cellular components (CC), molecular functions (MF) categories, and pathways using the ClusterProfiler R Package (Wang et al., 2022). The data obtained were visualized by using bioinformatics^8^ (Ding et al., 2021).

### Protein–protein interaction network analysis and identification of the hub genes

The AR/YPFS-related genes were input into the String database^9^ a protein interaction functional enrichment analysis website, to construct the Protein–protein interaction (PPI) network with a cutoff of high confidence of 0.7 (Silva et al., 2020). The top three genes were screened out as the hub genes by evaluating the interaction strength of each node and the average degree value (Hu et al., 2019).

### Ovalbumin-induced allergic rhinitis mice

Male BALB/C mice aged 6–8 weeks were purchased from Jinan Pengyue Experimental Animal Breeding Co., Ltd. (Shandong, China; License Number: SYXK(Lu) 2014-0007). All mice were raised in a specific pathogen-free level environment in the Research Building of Yuhuangding Hospital, Yantai. All experimental animals were fed with standardized sterile feed and water, which was sterilized by high-pressure steam and cooled to room temperature. All experimental animals were dissected after fasting for 12 h. All animal experiments were conducted in accordance with the National Institutes of Health Guide for the Care and Use of Laboratory Animals and approved by the Yantai Yuhuangding Animal Ethics Committee.

Ovalbumin-induced AR mice were induced through two stages: The basic sensitization stage and the stimulation stage. In the basic sensitization stage, 200 μl of a mixture comprising 40 μg OVA (Solarbio, Beijing, China) and 2 mg aluminum hydroxide (Thermo Scientific, Waltham, MA, United States) gel were injected intraperitoneally into the OVA-induced AR group seven times on days 1, 3, 5, 7, 9, 11, and 13. In the stimulation stage, mice received nasal drops comprising 20 μl of 5% OVA solution following atomization with 5% ovalbumin saline solution in a homemade simple atomizer for 30 min, from days 21 to 27 (Sun et al., 2012). The mice in the control group were treated with 0.9% sterilized normal saline in the same way. Nasal symptoms were assessed immediately after the last OVA stimulation for 30 min as the scratching nose frequency and the times of scratching nose per session (Zhang et al., 2020).

### YU-Pingfeng San decoction preparation and treatment

YPFS comprises three kinds of herbs, HQ (lot:20200617, Beijing Materia Medica Fangyuan Pharmaceutical Technology Co. Ltd., China), BZ (lot:20602, Haozhou City, Jing Wan Chinese medicine yinpian factory, China), and FF (lot:201001, Beijing Materia Medica Fangyuan Pharmaceutical Technology Co. Ltd., China) at a content ratio of 3:1:1. The three herbs were mixed, soaked in water for 1 h, and then decocted two times using an automatic decocting machine for 1.5 h each time. The decoctions (extracted by Beijing Tongrentang Co., Ltd., China) were filtered through double gauze to concentrate the filtrate to the final concentration of 1 g/ml and stored at 4°C until use (Bao et al., 2019).

YPFS was intragastrically administered to the AR mice in the YPFS group at a dose of 130 mg/20 g body weight for 14 days continuously (Wang et al., 2019). The same amount of saline was intragastrically administrated into mice in the OVA-induced AR group. On days 3, 6, 9, and 12, 5% OVA was dropped into the nasal cavity to maintain intranasal stimulation. After the last administration, the blood and nasal mucosa of the mice were obtained by dissection under a microscope (Möller-Wedel, Wedel, Germany).

### Enzyme-linked immunosorbent assay

The serum was separated from the upper layer of the blood after centrifugation for 20 min at 900 g. The concentrations of immunoglobulin E (IgE), interleukin (IL)-4, IL-5, IL-13, and interferon-gamma (IFN-γ) in mouse serum were detected using an Enzyme-linked immunosorbent assay (ELISA) kit (Jingmei Biotechnology, Guangzhou, China) according to the manufacturer’s instructions.

### Quantitative real-time reverse transcription PCR

Total RNA was extracted from mouse nasal mucosa and human peripheral blood mononuclear cells (PBMCs) using the Trizol reagent (SparkJade, Qingdao, China) and reverse transcribed into cDNA using a reverse transcription kit (Accurate Bio, Changsha, China). The qPCR step was performed using an SYBR Green qPCR Mix kit (SparkJade) in a StepOnePlus fluorescence quantitative PCR instrument (Applied Biosystems, Foster City, CA, United States). The expression levels of the genes encoding IL-4, IL-5, epidermal growth factor receptor (EGFR), mitogen-activated protein kinase 1 (MAPK1), and protein kinase B1 (AKT1) were detected. The relative expression level was calculated using the 2^–ΔΔCT^ method (Livak and Schmittgen, 2001). The primers for mouse *Egfr*, *Mapk1*, and *Akt1* and human IL4, IL5, EGFR, MAPK1, and AKT1 are listed in Supplementary Table 1.

### Molecular docking analysis

The interactions between active ingredients and the AR/YPFS-related genes were analyzed using Cytoscape.^10^ Ingredients involved in regulating immune inflammation were selected for molecular docking with the three hub genes decursin (Yang et al., 2009; Shehzad et al., 2018), anomalin (Khan et al., 2016, 2019), and wogonin (Kim et al., 2018). The three-dimensional chemical structures of candidate compounds were retrieved from PubChem (see text footnote 2) and the crystal structures of the target proteins were retrieved from the RCSB Protein Data Bank (RCSB PDB^11^). The eutectic ligands isolated from the receptor and the active pocket of each target were identified using AutodockTools-1.5.6 (Caliceti et al., 2019). Autodock Vina-1.1.2 software (Algethami et al., 2021) was used to dock the active ingredients with the putative target molecules and determine their free binding energy. PyMOL software was used to visually analyze the interaction and binding patterns of active ingredients (Agnew et al., 2021).

### Human peripheral blood mononuclear cells culture and decursin treatment

Ficoll (Solarbio) was used to isolate Human PBMCs according to the manufacturer’s instructions. The Human PBMCs were cultured in 10% fetal bovine serum and 90% Roswell Park Memorial Institute-1640 medium for 48 h. Then, decursin (MCE, Macau, China) was added to PBMCs at concentrations of 0, 5, and 10 μM, respectively, and incubated for 48 h.

### Statistical analysis

The experimental data were analyzed using GraphPad Prism 8 (GraphPad Inc., La Jolla, CA, United States) using unpaired *t*-tests, and *p*-value < 0.05 was considered statistically significant. The results are shown as the mean and standard deviation.

## Results

### Active components of YU-Pingfeng San and prediction of their targets

**FIGURE 1:**
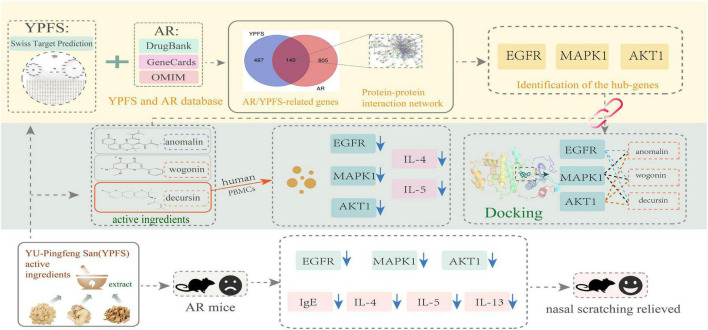
A workflow chart for treatment of allergic rhinitis with YU-Pingfeng San based on network pharmacology analysis and experimental verification.

**FIGURE 2:**
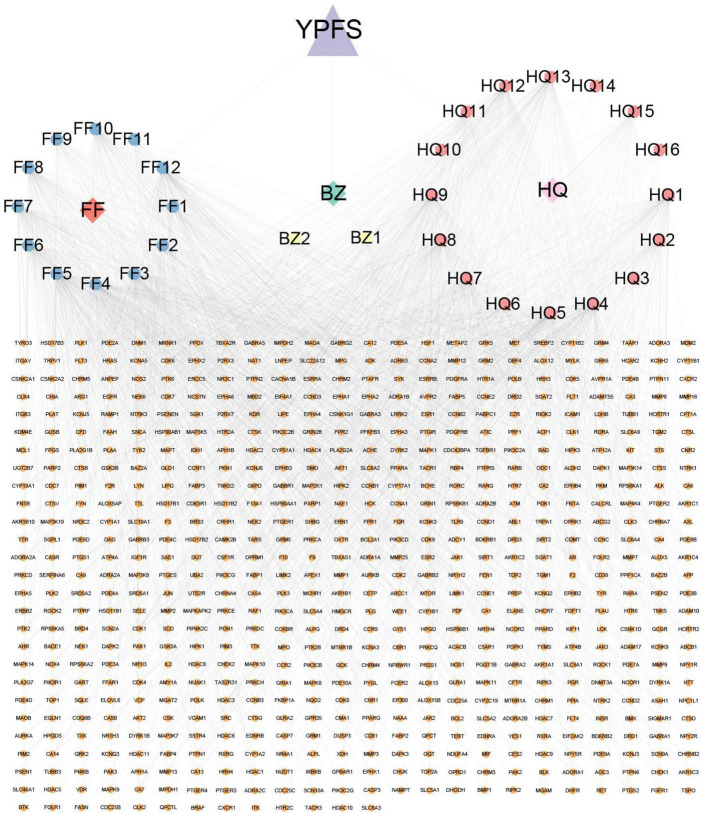
A network diagram was constructed with 3 active components, 30 active ingredients, and 637 target genes of these active ingredients using Cytoscape. The blue, yellow, and pink octagons represent the active ingredients of the three herbs in YPFS. FF1-12 are active ingredients of *Saposhnikoviae Radix* (Fangfeng, FF), BZ1-2 are active ingredients of *Atractylodis Macrocephalae Rhizoma* (Baizhu, BZ), and HQ1-16 are active ingredients of *Astragali Radix* (Huangqi, HQ). The full names of the corresponding active ingredients are listed in Supplementary Table 7. The orange circles represent YPFS-related genes, and the edges represent the interactions between the ingredients and the target genes. YPFS, YU-Pingfeng San.

The workflow chart for network pharmacology analysis and experimental verification is shown in Figure 1. Totally 20 active ingredients for HQ, 7 for BZ, and 18 for FF in YPFS were identified in the TCMSP. These 45 active ingredients of YPFS are shown in Supplementary Table 2. Among them, 30 active ingredients were predicted to have target proteins by Swiss target prediction, including 16 components for HQ, 2 for BZ, and 12 for FF. We found 637 potential drug targets for the 30 active ingredients (Supplementary Table 3). The interaction network among the three herbs, 30 active ingredients, and 637 target genes is shown in Figure 2.

### Screening of allergic rhinitis/YU-Pingfeng San-related genes

**FIGURE 3:**
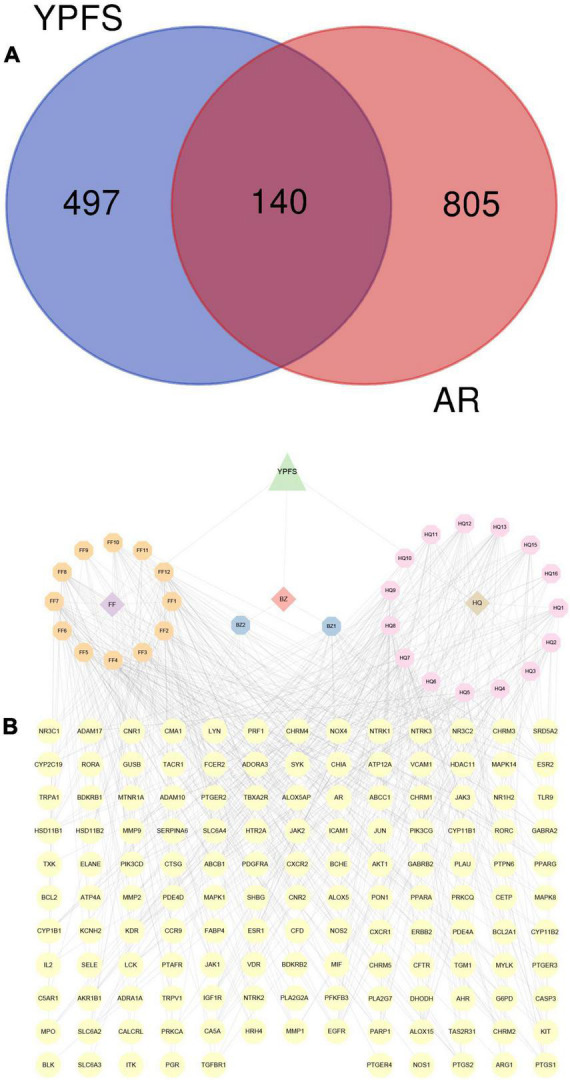
Screening for AR/YPFS-related genes. **(A)** Venn analysis of the common targets of YPFS and AR-associated genes. **(B)** The interaction network is constructed using ingredients and targets genes. The orange, blue, and pink octagons represent the active ingredients of the three herbs in YPFS. FF1-12 are active ingredients of *Saposhnikoviae Radix* (Fangfeng, FF), BZ1-2 are active ingredients of *Atractylodis Macrocephalae Rhizoma* (Baizhu, BZ), and HQ1-16 are active ingredients of *Astragali Radix* (Huangqi, HQ). The full names of the corresponding active ingredients are listed in Supplementary Table 7. The yellow circles represent the common targets of YPFS and AR-related genes, and the edges represent the interactions between the ingredients and the targets. YPFS, YU-Pingfeng San; AR, allergic rhinitis.

A total of 49 AR-associated target genes were obtained from the DrugBank database, 1,311 AR-associated target genes were obtained from the Genecards database, and 214 AR-associated target genes were obtained from the OMIM database. After combining the related target genes from the three databases and the removal of duplicates, a total of 945 AR-associated target genes were obtained (Supplementary Table 4). About 140 AR/YPFS-related genes (Supplementary Table 5) overlapped between the 945 AR-associated genes and the 637 YPFS target genes (Figure 3A). The interaction network among the three herbs, 29 active ingredients, and 140 AR/YPFS-related genes is shown in Figure 3B.

### Functional enrichment and pathway analysis

**FIGURE 4:**
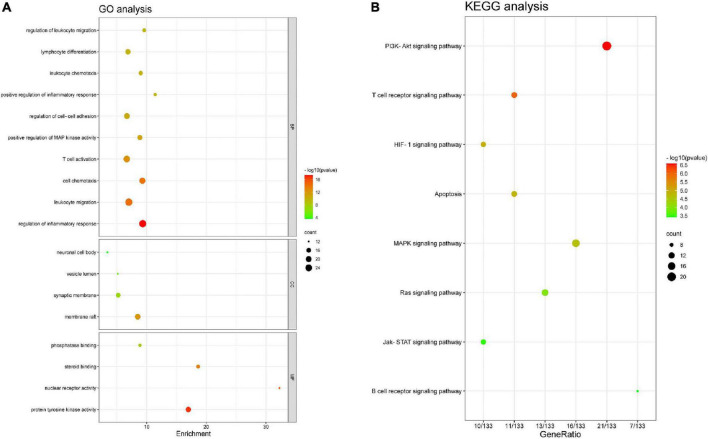
Enrichment analysis. **(A)** Gene ontology (GO) functional enrichment analysis. **(B)** Kyoto Encyclopedia of Genes and Genomes (KEGG) pathway enrichment analysis.

Functional enrichment analysis was performed on the 140 AR/YPFS-related genes using GO, which showed that these genes were mainly enriched in processes involved in immune regulation, such as regulation of inflammatory response, leukocyte migration and chemotaxis, regulation of leukocyte migration, lymphocyte differentiation, and T cell activation (Figure 4A). KEGG pathway enrichment analysis showed that these genes were enriched in certain immune and inflammatory-related pathways, such as the phosphatidylinositol-4,5-bisphosphate 3-kinase (PI3K)-AKT signaling pathway, the T cell receptor signaling pathway, the MAPK signaling pathway, the Janus kinase (Jak)-signal transducer and activator of transcription (STAT) signaling pathway, and the Ras signaling pathway (Figure 4B).

### Identification of the hub genes from among the allergic rhinitis/YU-Pingfeng San-related genes

**FIGURE 5:**
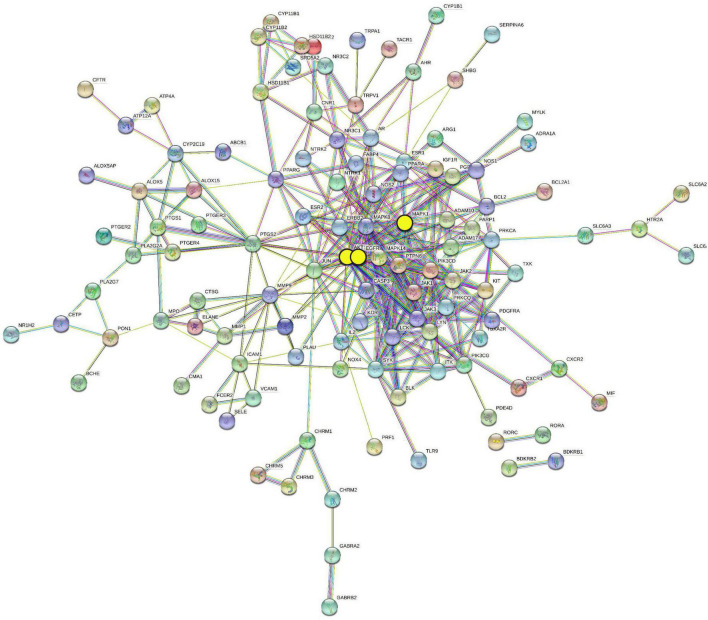
Protein–protein interaction (PPI) network of 140 AR/YPFS-related genes. The three yellow circles represent the selected genes: EGFR, MAPK1, and AKT1.

To understand the interaction regulation of the 140 AR/YPFS-related genes, we uploaded them to the String database to obtain a PPI network diagram (Figure 5). Using Cytoscape’s degree score analysis, we screened out three hub targets: EGFR, MAPK1, and AKT1 which had the strongest interaction scores of 32, 27, and 24, respectively. The degrees between target nodes are shown in Supplementary Table 6.

### YU-Pingfeng San relieved inflammatory reactions of ovalbumin-induced allergic rhinitis

**FIGURE 6:**
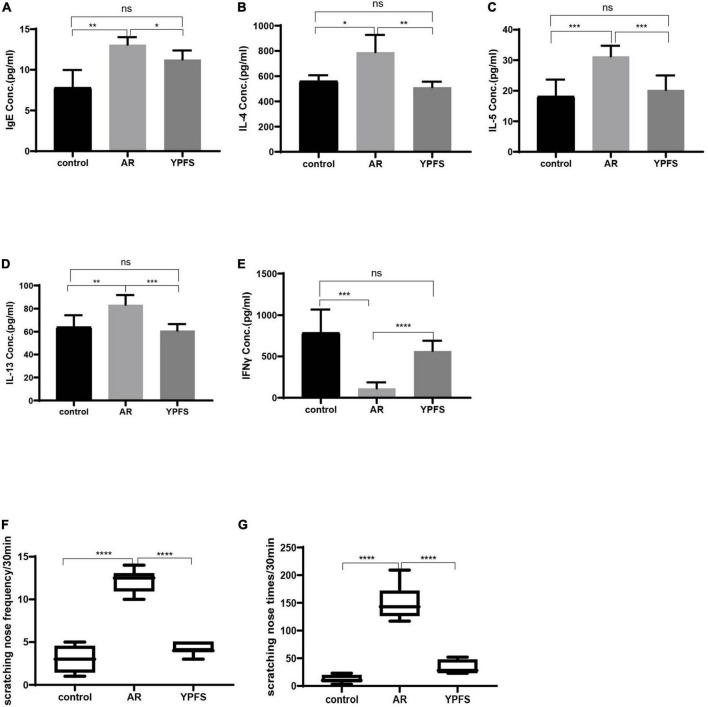
YPFS relieved inflammatory reactions of ovalbumin (OVA)-induced AR. Comparisons of IgE **(A)**, IL-4 **(B)**, IL-5 **(C)**, IL-13 **(D)**, and IFNγ **(E)** concentrations (Conc.) in the serum of mice. The frequency **(F)** and times **(G)** of nasal scratching scores in mice for 30 min in the control group, OVA-induced AR group, and YPFS group. *P < 0.05, **P < 0.01, ***P < 0.001, ****P < 0.0001; *n* = 6. YPFS, YU-Pingfeng San; AR, allergic rhinitis.

Compared with those in the control group, serum IgE, IL-4, IL-5, and IL-13 levels were significantly higher in the OVA-induced AR group (Figures 6A–D). The IFNγ level was significantly lower in the OVA-induced AR group than in the control group (Figure 6E). Through YPFS treatment, serum IL-5 and IL-13 levels decreased significantly (Figures 6A–D), and IFNγ levels increased significantly compared with those in the OVA-induced AR group (Figure 6E). Meanwhile, IgE, IL-4, IL-5, IL-13, and IFNγ showed no obvious change between the YPFS treatment group and the control group, which showed that YPFS treatment could rescue the levels of IgE, IL-4, IL-5, IL-13, and IFNγ to their normal levels. Compared with the control group, OVA modeling and YPFS treatment had no effect on the body weight of mice (Supplementary Figure 1).

Compared with those in the control group, the frequency and times of nasal scratching in the OVA-induced AR group mice increased significantly (Figure 6F), whereas the frequency and times of nasal scratching were reduced in the YPFS group (Figure 6G), showing that the AR symptoms were significantly relieved after YPFS treatment.

### YU-Pingfeng San reduced the expression of the hub genes in the nasal mucosa of ovalbumin-induced allergic rhinitis mice

**FIGURE 7:**
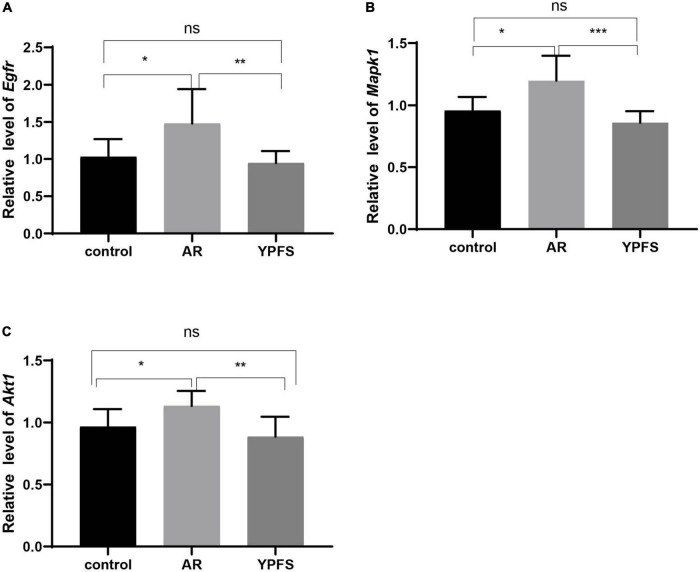
Relative expression levels of *Egfr* **(A)**, *Mapk1* **(B)**, and *Akt1* **(C)** in the nasal mucosa of mice in the control group, ovalbumin (OVA)-induced AR group, and YPFS group. *P < 0.05, **P < 0.01, ***P < 0.001; *n* = 6. YPFS, YU-Pingfeng San; AR, allergic rhinitis.

Compared with the control group, the expression levels of the hub genes *Egfr, Mapk1*, and *Akt1* in the nasal mucosa of the OVA-induced AR group were significantly increased, and no significant expression differences were found between the YPFS treatment group and the control group (Figures 7A–C). These results indicated that YPFS treatment could reduce the expression of the hub genes *Egfr, Mapk1*, and *Akt1* to their normal level.

### Identifying the docking sites between active ingredients and hub genes

**FIGURE 8:**
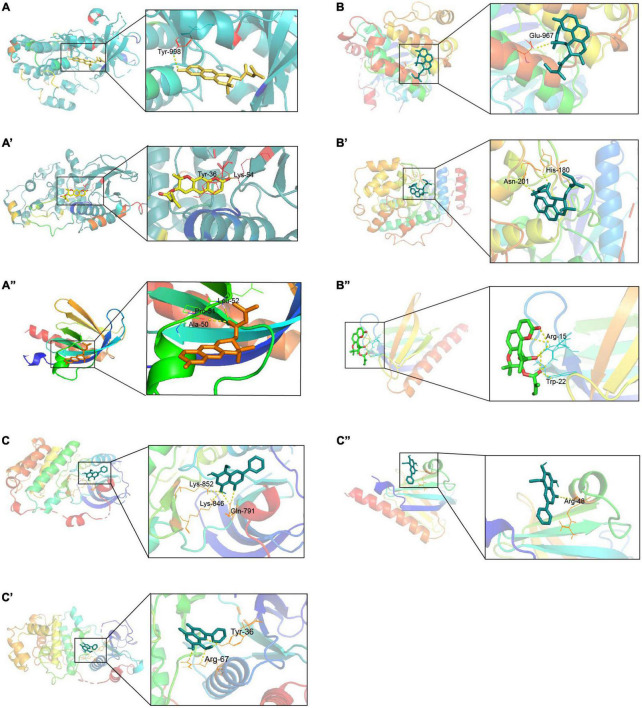
Docking sites of YPFS ingredients with target proteins. **(A)** Docking sites of EGFR and decursin. **(A’)** Docking sites of MAPK1 and decursin. **(A”)** Docking sites of AKT1 and decursin. **(B)** Docking sites of EGFR and anomalin. **(B’)** Docking sites of MAPK1 and anomalin. **(B”)** Docking sites of AKT1 and anomalin. **(C)** Docking sites of EGFR and wogonin. **(C’)** Docking sites of MAPK1 and wogonin. **(C”)** Docking sites of AKT1 and wogonin. EGFR PDB ID: 1XKK. MAPK1 PDB ID: 1PME. AKT1 PDB ID: 1H10.

Through the correlation analysis between active ingredients and the 140 AR/YPFS-related genes (Supplementary Table 7), three active ingredients, decursin, anomalin, and wogonin, were selected for molecular docking with EGFR, MAPK1, and AKT1. The results showed that decursin could bind to EGFR at Tyr998 (Figure 8A); to MAPK1 at Tyr36 and Lys54 (Figure 8A’); and to AKT1 at Ala50, Pro51, and Leu52 (Figure 8A”). Anomalin was identified to bind to EGFR at Glu967 (Figure 8B); to MAPK1 at Asn201 and His180 (Figure 8B’); and to AKT1 at Arg15 and Trp22 (Figure 8B”). Meanwhile, wogonin was identified to bind to EGFR at Lys842, Lys846, and Gln791 (Figure 8C); to MAPK1 at Tyr36 and Arg67 (Figure 8C’); and to AKT1 at Arg48 (Figure 8C”). These results showed that YPFS could target the hub proteins EGFR, MAPK1, and AKT1 via the active ingredients decursin, anomalin, and wogonin.

### Decursin decreased the expression of inflammatory factors in human peripheral blood mononuclear cells

**FIGURE 9:**
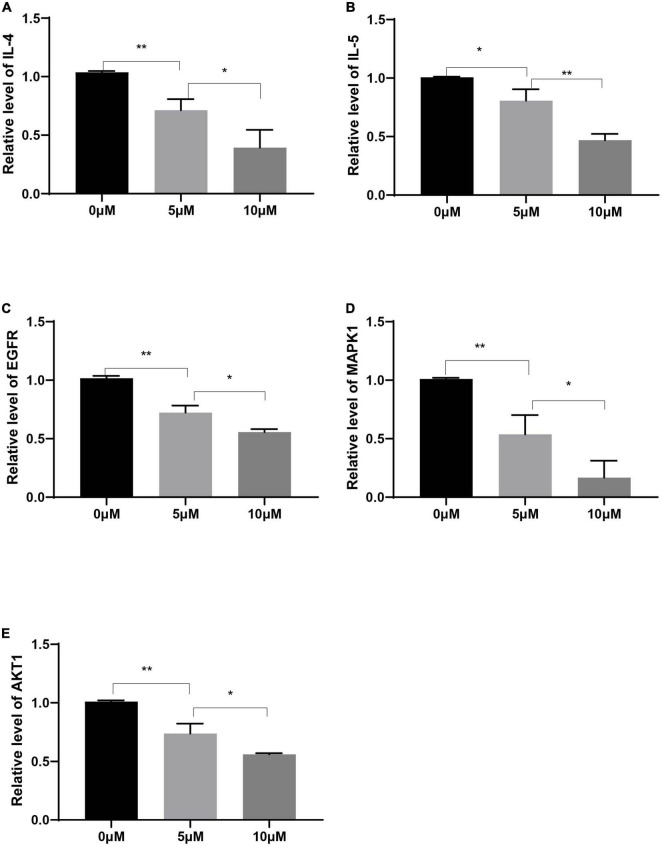
Relative expression levels of IL-4 **(A)**, IL-5 **(B)**, EGFR **(C)**, MAPK1 **(D)**, and AKT1 **(E)** in Human PBMCs treated with decursin at 0, 5, and 10 μM. *P < 0.05, **P < 0.01; *n* = 3.

Compared with those in the control group, the expression levels of IL-4 and IL-5 in Human PBMCs after decursin treatment were decreased in a dose-dependent manner (Figures 9A,B). Similarly, the expression levels of EGFR, MAPK1, and AKT1 in Human PBMCs decreased after decursin treatment (Figures 9C–E). These results showed that decursin could relieve the inflammatory reaction in AR by affecting EGFR, MAPK1, and AKT1 expression in Human PBMCs.

## Discussion

TCM formulations composed of multiple ingredients can affect multiple targets and pathways via complex mechanisms. YPFS is often used to treat AR in the clinic; however, its mechanism of action remains unclear. In this study, using network pharmacology analysis and experimental verification, the active ingredients and target genes of YPFS were identified for AR treatment. Based on the network pharmacology analysis, 30 active ingredients and 140 AR/YPFS-associated genes were screened from network databases. Through PPI network analysis of the 140 targets, three hub gene targets with the strongest interactions were found: AKT1, MAPK1, and EGFR, which are enriched in T-helper 1 (Th1) and 2 types (Th2) immune responses and T cell cytokine production. Through *in vivo* experiments, YPFS treatment was observed to relieve AR symptoms, reduce the expression levels of Th2 cytokines IL-4, IL-5, and IL-13, and increase the expression level of the Th1 cytokine IFNγ in serum. Meanwhile, the expression levels of MAPK1, EGFR, and AKT1 were reduced by YPFS treatment. Through a molecular docking approach of ingredients and targets and *in vitro* cell experiments, decursin was found to have the most potential as the bioactive ingredient in YPFS to relieve the Th2 inflammatory response of AR by decreasing the expression of the hub genes MAPK1, EGFR, and AKT1.

In AR, allergens stimulate nasal epithelial cells to secrete inflammatory factors acting on innate lymphoid cells type 2 (ILC2) to produce the type 2 cytokines IL-5 and IL-13 and also acting on dendritic cells (DCs). DCs present antigens to resident lymph nodes to activate CD4^+^ T cell differentiation to Th2 cells, which produce the cytokines IL-4, IL-5, and IL-13, thus inducing a classical type 2 inflammatory response (Peng et al., 2020). In our study, the 140 AR/YPFS-related genes were enriched in those biological processes regulating AR pathogenesis such as lymphocyte chemotaxis, differentiation and migration, and T cell activation, further showing that these genes could play important roles in AR regulation through mediating lymphocyte activities.

AR is an allergic disease of the nasal mucosa caused by the imbalance of differentiation of T cells. YPFS is recommended for the management of AR by the Chinese medicine clinical practice guidelines and can relieve the clinical symptoms of AR. However, how YPFS functions is still not clear. YPFS could impair the inflammatory response by reducing thymic stromal lymphopoietin (TSLP), tumor necrosis factor-alpha (TNFα), and IL-6 production (Shen et al., 2014; Liu et al., 2017). YPFS also decreased eosinophils and the IgE content in serum and reduced IL-4 and IFNγ in lung tissue in OVA-induced allergic asthmatic mice (Chen et al., 2021). Moreover, YPFS has been reported to impair the inflammatory response by regulating inflammatory factor production (Shen et al., 2014), tight junctions (Zheng et al., 2019), and epithelial-mesenchymal transition (Yao et al., 2019) in epithelial cells. However, little is known about how YPFS regulates the Th2 cell response and Th2-type inflammatory factor production in AR. In the present study, the levels of Th2-type inflammatory factors IL-4, IL-5, and IL-13 were markedly reduced by YPFS treatment in OVA-induced AR mice, suggesting that YPFS could relieve AR symptoms by inhibiting the Th2 cell response. Meanwhile, we also found that IFNγ production was increased by YPFS. These results indicated that YPFS preferentially increases the Th1 cell response and decreases the Th2 cell response.

KEGG pathway enrichment analysis showed that 140 AR/YPFS-related genes were enriched in pathways involved in regulating AR, such as the PI3K/AKT pathway, the hypoxia-inducible factor 1 alpha (HIF1α) signaling pathway, and the MAPK, RAS, and JAK/STAT signaling pathways. The PI3K/AKT signaling pathway is involved in a variety of AR inflammatory responses, including Th2-mediated inflammation (Zeng et al., 2018), the percentage of regulatory T cells (Tregs), and IL-10 and TGF-β1 expression (Zeng et al., 2019), the expression of ILC2 cell transcription factors and type II cytokines (Zeng et al., 2020); mast cell activity suppression (Lin et al., 2015); and vascular endothelial growth factor (VEGF) and fibroblast growth factor 2 (FGF-2) expression in nasal epithelial cells (Chen et al., 2016). The level of HIF-1α is increased in patients with AR, and HIF-1α accumulation is critical for sustaining human allergic effector cell survival and function (Cheng et al., 2016; Kou et al., 2018). HIF-1α deficiency decreased inflammatory responses and symptoms caused by AR (Niu et al., 2020). HIF-1α inhibitors or antagonists could induce antiallergic effects by decreasing both local and systemic Th2 cytokine (IL-4 and IL-5) production, IgE production, and eosinophil infiltration into the nasal mucosa in an AR model (Mo et al., 2014; Wang et al., 2016). MAPK and JAK/STAT signaling pathways play key roles in the proliferation, differentiation, and production of inflammatory cells and are involved in the activation of AR (Yang et al., 2021). After blocking the MAPK or JAK/STAT signaling pathways, the symptoms of rhinitis in AR mice were reduced, and the aggregation of inflammatory cells in the epithelial cells of the nasal mucosa and vascular area was decreased (Howell et al., 2018; Paiva Ferreira et al., 2021). These observations show that the pathways play important roles in AR regulation, and indicate the reliability of the 140 selected AR/YPFS-related genes.

Three hub genes, EGFR, MAPK1, and AKT1, were identified through PPI analysis and Cytoscape analysis. Accumulating studies have shown that EGFR, MAPK1, and AKT1 participate in AR regulation. EGFR was significantly elevated in patients with AR (Matovinovic et al., 2003), and the activation of EGFR was likely to drive airway inflammation and epithelial cytokine production (Daines et al., 2020). EGFR inhibitors have been shown to inhibit OVA-induced metaplasia, mucus production, and eosinophilic/neutrophil infiltration in rat nasal epithelial goblet cells, and inhibition of the EGF signaling pathway activity alleviates nasal mucosal injury (Shimizu et al., 2018). The results of the present study confirmed that after YPFS treatment of AR mice and EGFR reduction, the expression of inflammatory factors in the OVA-induced AR group decreased, and rhinitis symptoms were alleviated, suggesting that EGFR reduction benefits AR treatment. MAPK1 and AKT1 participate in AR regulation via the MAPK and PI3K/AKT signaling pathways, respectively. MAPK1 was also identified as the core gene of YPFS targets in a study by Yang et al. based on network pharmacology (Yang et al., 2021). In our study, the elevated expression of MAPK1 and AKT1 were reversed by YPFS treatment in the AR mice. Thus, YPFS could alleviate the AR inflammatory response and symptoms by reducing the expression levels of EGFR, MAPK1, and AKT1. Gene enrichment analysis showed that EGFR, MAPK1, and AKT1 are involved in the HIF-1α pathway, indicating that YPFS could also alleviate AR responses by regulating the HIF-1α pathway.

The compounds decursin, anomalin, and wogonin, screened from YPFS, can potentially bind to EGFR, MAPK, and AKT and inhibit their expression. Decursin has been reported to be associated with inflammatory immunity (Shehzad et al., 2018). A novel (S)-(+)-decursin derivative reduced ovalbumin-specific immunoglobulin E (IgE) levels in an OVA-induced mouse model of asthma and alleviated lung inflammation in mice, and thus might be an effective therapeutic agent for allergic airway diseases (Yang et al., 2009). Studies have found that decursinol angelate inhibits the expression of pro-inflammatory cytokines, such as IL-1β and IL-6, NADPH oxidase (NOX), and inducible nitric oxide synthase (iNOS) in cells. It has the potential to inhibit macrophage polarization and inflammation by blocking the activation of pro-inflammatory signals (Islam et al., 2018). Decursin analogs also inhibit inflammation by downregulating nuclear factor kappa B (NF-κB) and STAT1 (Lee et al., 2021). Anomalin significantly inhibited the production of pro-inflammatory mediators and significantly reduced the activation of MAPK and AKT proteins in macrophages (Khan et al., 2019). Studies have shown that anomalin inhibits the levels of inflammatory cytokines through the inactivation of NF-κB, nuclear factor erythroid 2-related factor 2 (Nrf2), and MAPK signaling pathways (Khan et al., 2016). Wogonin produced an antiallergic effect in AR mouse models by reducing eosinophil infiltration and the Th2 cytokines IL-4, IL-5, and IL-13 in the serum and nasal mucosa (Shin et al., 2014; Kim et al., 2018). Wogonin can also reduce allergic airway inflammation *in vivo* by reducing the number of eosinophils, increasing eosinophil apoptosis, reducing airway mucus production, and reducing airway hyperresponsiveness. Studies have shown that wogonin might play a role in the treatment of allergic diseases by regulating Th1/Th2 cytokine imbalance and histamine release in mast cells (Bui et al., 2017). These studies support our results that decursin, anomalin, and wogonin might be the key active ingredients in YPFS for AR treatment.

## Conclusion

In summary, we identified the active ingredients in YPFS and 140 AR/YPFS-related genes through network pharmacology analysis. *In vivo* experiments showed that YPFS reduced the IgE level in serum, decreased the production of Th2-type cytokines, and relieved AR symptoms. Meanwhile, EGFR, MAPK1, and AKT1 were found to be downregulated by YPFS in OVA-induced AR mice, and be targeted by active ingredients decursin, anomalin, and wogonin from YPFS through molecular docking prediction. These results showed that YPFS could relieve the AR inflammatory response and symptoms mainly via active ingredients decursin, anomalin, and wogonin targeting and inhibiting EGFR, MAPK1, and AKT1, which may provide novel ingredients for AR treatment. However, these results should be further verified using more detailed molecular mechanism studies, contributing to the *in-depth* research and application of traditional Chinese medicines.

## Data availability statement

The original contributions presented in this study are included in the article/Supplementary material, further inquiries can be directed to the corresponding authors.

## Ethics statement

The studies involving human participants were reviewed and approved by Yantai Yuhuangding Animal Ethics Committee. The patients/participants provided their written informed consent to participate in this study. The animal study was reviewed and approved by Yantai Yuhuangding Animal Ethics Committee.

## Author contributions

YY and YL designed and directed the study. ZL and QS organized the public data and wrote the manuscript. ZL, XL, XS, QS, and ZS performed the experimental work and analyzed the data. FS, CL, and QS took charge of data visualization. XS, YZ, and YL revised the manuscript. All authors contributed to the article and approved the submitted version.

## Footnotes

1
https://www.tcmsp-e.com/

2
https://pubchem.ncbi.nlm.nih.gov/

3
http://www.swisstargetprediction.ch/

4
https://www.drugbank.com/datasets

5
https://www.genecards.org/

6
https://omim.org/

7
http://bioinformatics.psb.ugent.be/webtools/Venn/

8
http://www.bioinformatics.com.cn/

9
https://cn.string-db.org/

10
https://cytoscape.org/

11
https://www.rcsb.org/

## Abbreviations

YPFS: YU-Pingfeng San
AR: allergic rhinitis
EGFR: epidermal growth factor receptor
MAPK1: mitogen-activated protein kinase 1
AKT1: protein kinase B1
TCMs: traditional Chinese medicines
HQ: *Astragali Radix*
BZ: *Atractylodis Macrocephalae Rhizoma*
FF: *Saposhnikoviae Radix*
GO: Gene ontology
KEGG: Kyoto Encyclopedia of Genes and Genomes
OVA: ovalbumin
IL: interleukin
IgE: immunoglobulin E
IFN-γ: interferon-gamma
PBMCs: peripheral blood mononuclear cells
