# Supplementary figures and images for: Network pharmacology analysis and experimental verification reveal the mechanism of the traditional Chinese medicine YU-Pingfeng San alleviating allergic rhinitis inflammatory responses
Source: Front Plant Sci. 2022 Aug 9;13:934130. doi: 10.3389/fpls.2022.934130 (PMC9396374; doi:10.3389/fpls.2022.934130)

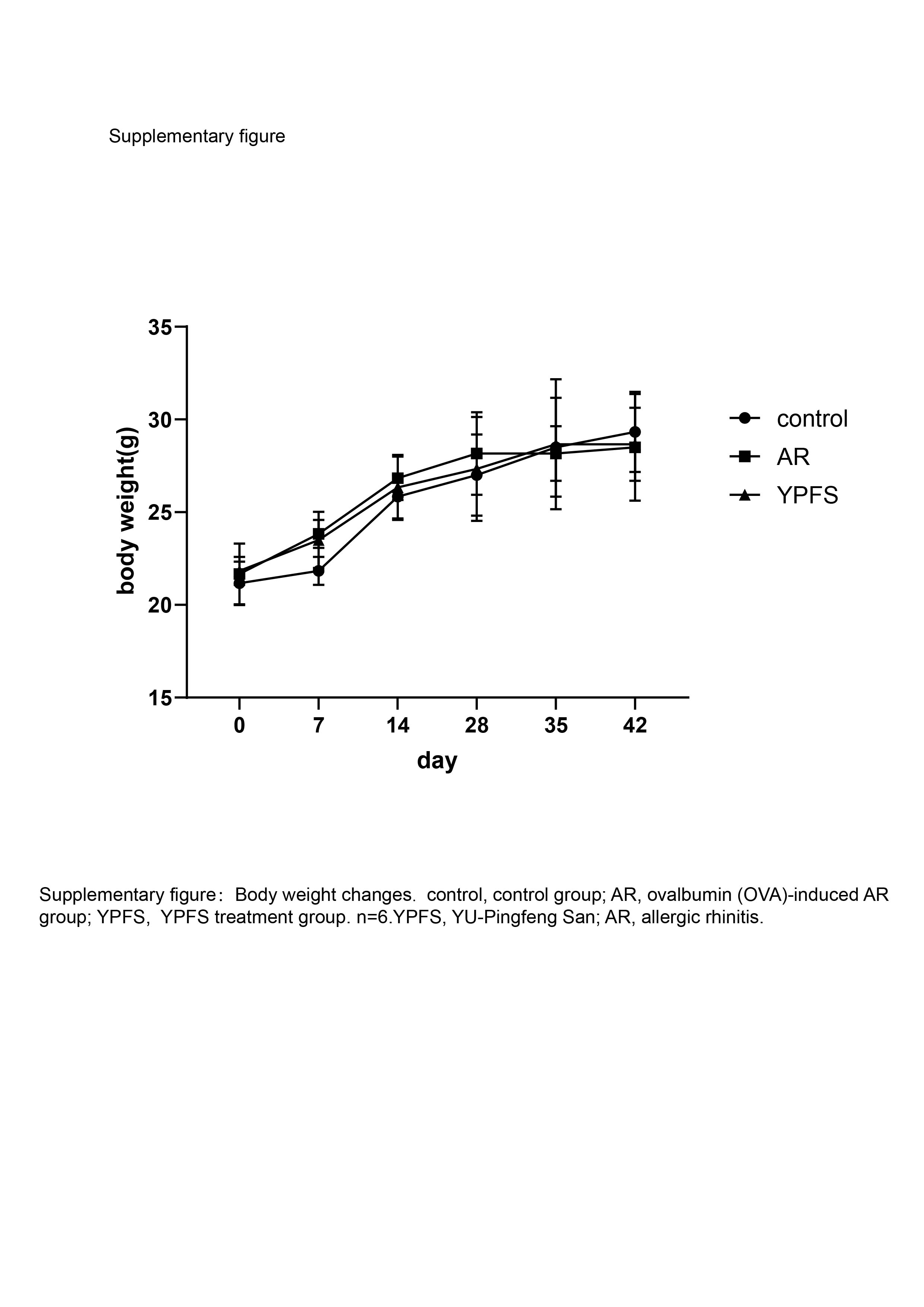

Supplement: Supplementary file 8 [file Image_1.JPEG]
